# Solvothermal Synthesis of Cu_2_ZnSnSe_4_ Nanoparticles and Their Visible-Light-Driven Photocatalytic Activity

**DOI:** 10.3390/nano14131079

**Published:** 2024-06-24

**Authors:** Rodrigo Henríquez, Paula Salazar Nogales, Paula Grez Moreno, Eduardo Muñoz Cartagena, Patricio Leyton Bongiorno, Pablo Zerega Garate, Elena Navarrete-Astorga, Enrique A. Dalchiele

**Affiliations:** 1 Instituto de Química, Facultad de Ciencias, Pontificia Universidad Católica de Valparaíso, Casilla 4059, Valparaíso 2340000, Chile; paula.salazar@pucv.cl (P.S.N.); paula.grez@pucv.cl (P.G.M.); eduardo.munoz.c@pucv.cl (E.M.C.); patricio.leyton@pucv.cl (P.L.B.); pablo.zerega@pucv.cl (P.Z.G.); 2 Laboratorio de Materiales y Superficie, Departamento de Física Aplicada I, Universidad de Málaga, 29071 Málaga, Spain; enavarrete@uma.es; 3 Instituto de Física, Facultad de Ingeniería, Herrera y Reissig 565, C.C. 30, Montevideo 11000, Uruguay; dalchiel@hotmail.it

**Keywords:** Cu_2_ZnSnSe_4_, nanoparticles, solvothermal, photocatalyst, CR

## Abstract

Cu_2_ZnSnSe_4_ (CZTSe) nanoparticles (NPs) were successfully synthesized via a solvothermal method. Their structural, compositional, morphological, optoelectronic, and electrochemical properties have been characterized by X-ray diffraction (XRD), Raman spectroscopy, X-ray photoelectron spectroscopy (XPS), Field-emission scanning electron microscopy (FE-SEM), transmission electron microscope (TEM), UV–vis absorption spectroscopy, and electrochemical impedance spectroscopy (EIS) techniques. Porosimetry and specific surface area in terms of the Brunauer–Emmett–Teller (BET) technique have also been studied. XRD indicates the formation of a polycrystalline kesterite CZTSe phase. Raman peaks at 173 and 190 cm^−1^ confirm the formation of a pure phase. TEM micrographs revealed the presence of nanoparticles with average sizes of ~90 nm. A BET surface area of 7 m^2^/g was determined. The CZTSe NPs showed a bandgap of 1.0 eV and a p-type semiconducting behavior. As a proof of concept, for the first time, the CZTSe NPs have been used as a visible-light-driven photocatalyst to Congo red (CR) azo dye degradation. The nanophotocatalyst material under simulated sunlight results in almost complete degradation (96%) of CR dye after 70 min, following a pseudo-second-order kinetic model (rate constant of 0.334 min^−1^). The prepared CZTSe was reusable and can be repeatedly used to remove CR dye from aqueous solutions.

## 1. Introduction

Among the emerging inorganic photovoltaic materials, the family of kesterite semiconductor materials has raised considerable expectations for the development of low-cost and high-efficiency thin-film solar cells [1]. The kesterite family includes quaternary and quinary compounds such as Cu_2_ZnSnS_4_ (CZTS), Cu_2_ZnSnSe_4_ (CZTSe), and their alloys (Cu_2_ZnSn(S_x_,Se_1−x_)_4_ (CZTSSe), where 0 ≤ x ≤ 1) [2,3,4,5]. The kesterite constituent elements are less toxic and are the more usual elements of copper, zinc, and sulfur, which are the 26th, 25th, and 17th most abundant elements in the Earth’s crust, respectively [2,6,7,8,9,10]. All these kesterite semiconductor materials exhibited outstanding optoelectronic properties such as broad optical absorbance over the entire visible region with high absorption coefficient (>10^4^ cm^−1^) [6,7,9,11] and optimal optical bandgap (0.9–1.6 eV), allowing the material to harvest maximum photons [12], good photo-stability [13], and multi-dimensional symmetric carrier transport [11,14]. Moreover, it is possible to tune their optoelectronic properties (i.e., optical bandgap, E_g_) through modulation of the kesterite phase composition (S/Se ratio) [4]. In fact, copper zinc tin sulfide, CZTS, has the highest band gap (1.4–1.6 eV), while replacing sulfur with selenium in CZTSe leads to a lower band gap (0.8–1.0 eV) [4]. Then, the kesterite material bandgap can be tuned between an optimal region from 1.0 to 1.5 eV, which makes it also interesting for multijunction applications [5,11,15].

In the last decades and, unfortunately, until today, the organic wastewater produced in modern industrial production has caused and continues to cause serious environmental pollution problems [12,16,17]. Particularly because of their toxicity, mutagenicity, carcinogenicity, and slow biodegradability, residual dyes from the textile industry cause a serious risk to the health of humans and animals [12,18]. In fact, these dyes cause serious respiratory issues, damaging DNA and causing cancer in humans [12]. Moreover, aquatic life is impacted by prolonged dye exposure in water, which poses a major risk to the ecosystem [12]. It must be pointed out that one-fifth of water pollution comes from textile dyes [19]. It is, therefore, essential and imperative to have efficient treatment processes for the safe disposal of those dye-contaminated effluents [12,16,17,18]. In this way, in the last three decades, several research studies have focused on photocatalysis on semiconductor surfaces, which play a predominant role in the water remediation process [12,16,20]. Semiconductor-based photocatalytic reactions are a practical class of advanced oxidation processes (AOPs) to address the remediation of environmental pollutants [21]. The semiconductor-based photocatalytic process is anticipated to be the preferred method for treating wastewater among the different AOPs because of its superior characteristics, such as its environmentally friendly nature, simple manufacturing, complete mineralization capability, and reusability [20,21]. Through the photocatalytic process, some toxic and harmful organic macromolecular substances are degraded, converting them into non-toxic and harmless small molecules so as to achieve the purpose of environmental protection [17,20]. Photocatalytic degradation has been utilized to treat contaminants ranging from dyes (methylene blue, methyl orange, Congo red, and rhodamine B), chemical precursors, and pharmaceuticals (tetracycline, enrofloxacin, diclofenac), to diverse organic and inorganic waste [17,20,22]. In recent decades, the semiconducting materials that are essential to the water photocatalytic remediation process have been the subject of numerous studies [12,16,17,20]. Among them, wide bandgap semiconducting materials such as TiO_2_ and ZnO are used as traditional photocatalysts due to their stable and non-toxic nature [12,16,17,20]. However, the limitations of those traditional semiconducting photocatalysts are very poor absorption over the visible region (can only absorb ultraviolet (UV) light, highlighting that UV light accounts for less than 4% of the solar spectrum) and exhibit a high carrier charge recombination which hinders the photocatalytic activity [12,16,17,20]. To tackle these restrictions, the main focus of research is on visible-light-driven photocatalysts with narrow bandgap semiconductors exhibiting optical absorbance throughout the whole visible region [12,16]. Due to their adequate nature and outstanding optoelectronic properties (as has been said and described above), researchers are encouraged to work on kesterite semiconductor materials as a suitable candidate in the field of semiconductor photocatalysis [12,23,24]. In fact, in recent years, the CZTS semiconducting photocatalyst compound has been well documented and intensively studied in water remediation of organic pollutants and industrial wastes [13,25,26,27,28,29,30,31,32,33,34]. However, until now, there have been few works reporting the employment of the CZTSe compound as a semiconducting photocatalytic material. Kush et al. reported that 0.004 mM of methylene blue (MB) and Rhodamine B (RhB) solutions were degraded under indoor illumination above 90% within 35 and 120 min, respectively [35]. Han et al. reported 90% degradation of RhB (10 mg L^−1^) with CZTSe under indoor illumination within 120 min [16]. Prasanna et al. reported that 70% degradation of a highly concentrated MB (0.02 mM) solution occurred within 120 min with nanocrystalline CZTSe material under indoor illumination [12]. To our knowledge, there are no results available on CZTSe-based photocatalytic studies for the degradation of Congo red (CR) azo dye, which is one of the motivations of the present work. 

Different methods of synthesis have been proposed and used for the preparation of the quaternary CZTSe material, including physical methods and wet chemical routes [1,36,37,38]. Recently, CZTSe nanocrystals have been prepared by different wet chemical solution-based routes, i.e., high temperature arrested precipitation, solvothermal, hydrothermal, colloidal hot injection route, automated continuous-flow process, microwave-assisted, and others [1,6,12,36,37,38,39,40,41]. Among them, as an important method for wet chemistry, featuring low temperature, simplicity, good yield, convenient handling, inexpensive equipment, and controllable uniform particle size and regular morphology, the solvothermal method has been widely employed for the preparation of quaternary CZTSe nanocrystalline samples [1,6,36,37,40,41,42,43,44,45,46,47]. Moreover, in the solvothermal method, the growth conditions, such as solvent, temperature, duration, and precursor sources, can be varied to tune the phase, morphology, and shape of the CZTSe particles [36,43,46,47]. In the present study, the solvothermal synthesis of CZTSe nanoparticles was performed using ethylenediamine as the solvent. This method was carried out at low temperatures and did not require organometallic or toxic precursors. 

As a proof of concept, the synthesized nanoparticulate Cu_2_ZnSnS_4_ samples have been used as a visible-light-driven photocatalyst for Congo red (CR) azo dye degradation, showing high photocatalytic activity and stability.

## 2. Materials and Methods

### 2.1. Solvothermal Synthesis

The synthesis of CZTSe nanoparticles (CZTSe-NPs) was performed employing an ethylenediamine solution containing a mixture of 1 mmol CuCl_2_, 2 mmol ZnCl_2_ (Sigma-Aldrich P.A., St. Louis, MI, USA), 2 mmol SnCl_2_ (Winkler P.A.), and 4 mmol Se elemental (Merck P.A., Rahway, NJ, USA) in a hydrothermal reactor at 180 °C for 72 h. The obtained CZTSe-NPs were washed with 1:1 ethanol/water and centrifuged for 15 min at 4500 rpm (DLAB model DM0412, Beijing, China). Finally, the CZTSe nanoparticles were thermally treated for 1 h at 400 °C in an Ar atmosphere.

### 2.2. Characterization of the CZTSe-NPs

CZTSe-NPs were characterized by different techniques that provided information about their structural and chemical composition and morphological, optical, and optoelectronic properties. These characterizations were performed as previously reported by our group [33] (see also Supplementary Material SI).

The surface area of the samples was determined using the Micromeritics 3FLEX instrument (Haan, Germany) for the volumetric adsorption–desorption isotherm of N_2_ at −196 °C, using 0.05 g of each sample, which were degassed for 6 h at 180 °C under vacuum using a Micromeritics SmartVacPrep instrument (Norcross, GA, USA). The surface area was determined using the Brunauer–Emmett–Teller (BET) equation in the relative pressure range of 0.05 ≤ P/P_0_ ≤ 0.25. The total pore volume was defined as the pore volume of a single point at P/P_0_ = 0.99. The average pore diameter (d_p_) was obtained from the desorption branch using the Barrett–Joyner–Halenda (BJH) method. 

For the electrochemical studies, supported nanoparticulated CZTSe working electrodes were prepared. To this end, the addition of CZTSe thin layers onto previously cleaned FTO/glass substrates (1.5 × 1.0 cm^2^) was performed using a drop-casting procedure. So, a previously ultrasonicated (for half an hour) nanoparticulate CZTS suspension (25–30 mg of CZTSe in 4–5 mL of ethanol) was drop-casted onto the surface of the FTO/glass substrate (drop by drop until completing a total of 10 drops), using a pipette. Afterward, these layers were dried in an Ar atmosphere under heat (at 60 °C) for 4 h, obtaining the CZTSe/FTO glass substrate electrode.

For the electrochemical characterization of the CZTSe nanoparticles, cyclic voltammetry and electrochemical impedance spectroscopy (EIS) were performed in a three-electrode conventional cell with the CZTSe/FTO as the working electrode, Ag/AgCl (3 M KCl) as the reference electrode, and a platinum wire as the auxiliary electrode, all immersed in a 0.1M Na_2_SO_4_ solution as the supporting electrolyte. In all cases, the measurements were carried out at room temperature, exposing an area of 1.5 cm^2^ to the supporting electrolyte.

The cyclic voltammetry was performed starting anodically from open circuit potentials (ac. 0.03 V vs. Ag/AgCl) scanning between −0.5 V and 0.1 V at a potential scan rate of 0.02 V s^−1^.

The Nyquist and Bode spectrums were obtained from an AUTOLAB model PGSTAT 302 potentiostat/galvanostat (Herisau, Switzerland) with a FRA2 impedance module at open circuit potential conditions in a frequency range of 100 kHz to 100 mHz. Mott Schottky measurements were performed employing a FRA ZAHNER model ZENNIUM PP211 potentiostat/galvanostat (Kronach, Germany) developed in the region of potentials without faradaic processes indicated by the cyclic voltammetry plot. A 10 mV AC voltage was employed as the perturbation amplitude. The response was analyzed considering a parallel electrical circuit. 

### 2.3. Photodegradation of CR Azo Dye and Photocatalyst Regeneration Studies

To establish the photodegradation of dye and the regeneration of the CZTSe-NP photocatalyst, studies were carried out employing a 50 mL solution of 0.04 mM Congo red azo dye, to which 35 mg of Cu_2_ZnSnSe_4_ nanoparticles were added. The studies were performed as previously reported by our group [33] (see also Supplementary Material SI).

## 3. Results

### 3.1. Structural, Morphological, and Surface Chemical Study

To ascertain the structural and morphological characteristics, as well as the chemical composition of the surface, X-ray diffraction (XRD), Raman spectroscopy, X-ray photoelectron spectroscopy (XPS), field emission-scanning electron microscopy (FE-SEM), and high-resolution transmission electron microscopy (HR-TEM) have been used to characterize the prepared CZTSe samples.

**Figure 1:**
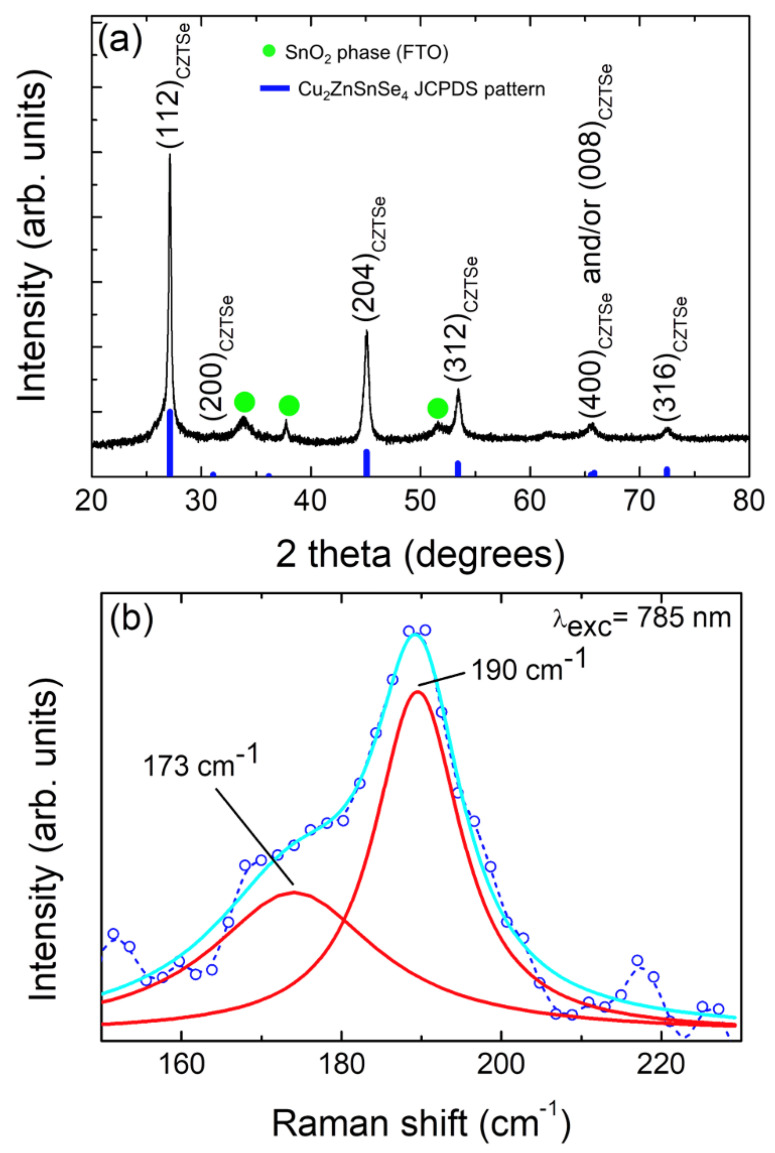
(**a**) X-ray diffraction pattern of a typical nanoparticulate Cu_2_ZnSnSe_4_ sample. Diffraction planes are indicated for the Cu_2_ZnSnSe_4_ phase (CZTSe(hkl)). (**b**) Raman spectrum of nanoparticulate Cu_2_ZnSnSe_4_ nanopowder sample excited with a wavelength of 785 nm. The dominant emissions are fitted using two Lorentz functions as indicated. The cyan line is the fit line, while the red lines represent each individual component. The original spectrum is represented by a blue dash line plus small blue open circles.

In a particular way, to further characterize the crystalline quality and study the nanoscale crystalline structure of CZTSe samples, the XRD patterns and Raman spectra of these samples have been analyzed. Figure 1 shows a typical diffraction pattern of a solvothermally grown nanoparticulate Cu_2_ZnSnSe_4_ sample. For comparison, the XRD pattern of the standard tetragonal crystalline structure of Cu_2_ZnSnSe_4_ (kesterite) (Space Group I42m, a = 5.6930 Å, b = 5.6930 Å, and c = 11.3330 Å, alpha = beta = gamma = 90°), JCPDS pattern #00-052-0868, is also provided [48]. The XRD pattern exhibits five main intense diffraction peaks at 2θ = 27.1°, 2θ = 45.0°, 2θ = 53.4°, 2θ = 65.6°, and 2θ = 72.5° ascribed to the (112), (204), (312), (400)/(008), and (316) diffraction planes of the tetragonal crystalline structure of CZTSe, respectively. In addition, a faint diffraction peak at 2θ = 31.1° can be seen, which can also be ascribed to the (200) diffraction plane of the kesterite CZTSe phase. Then, the XRD results indicate that the formed kesterite CZTSe phase is well-defined, and the samples are polycrystalline. The presence of very small impurity diffraction peaks corresponding to SnO_2_ can be appreciated (see Figure 1), indicating the presence of a minor SnO_2_ impurity secondary phase. From the XRD pattern, it is observed that the CZTSe sample is polycrystalline in nature. Moreover, the broadening of the CZTSe diffraction peaks demonstrates the nanocrystalline character of these samples. The average crystallite size was calculated from the full width at half maximum (FWHM) of the XRD peaks by using the well-known Scherrer formula [49,50] (please see SI, Equation (S2)).

When the term “crystallite size” is used, we refer to the dimensions of the coherent diffracting domain. The average crystallite size evaluated from the (112) CZTSe diffraction peak was ~35 nm, inferring its nanocrystalline character. However, it is worth mentioning that due to the overlapping between characteristic XRD peaks of CZTSe and the diffraction peaks of Cu_x_Se, Cu_2_SnSe_3_, and ZnSe XRD patterns [4,8,42,51], the coexistence of these phases cannot be completely excluded based solely on XRD analysis [4,8,42,51]. Therefore, to confirm the structural properties of the CZTSe nanocrystalline samples and provide a more comprehensive understanding, Raman spectroscopy has been used as a complementary tool to distinguish and ensure the formation of the pure Cu_2_ZnSnSe_4_ phase. To differentiate various phases, the first-order Raman spectra in the 150–230 cm^−1^ wavenumber region (see Figure 1b) have been deconvoluted by the least square Lorentz fitting procedure. Figure 1b shows the Lorenz fitting result of the Raman spectrum obtained at room temperature and for an excitation wavelength of 785 nm from the CZTSe sample. As can be appreciated, the multiple peak fitting revealed two peaks centered at 173 and 190 cm^−1^. All these peaks are attributed to and are characteristics of the kesterite Cu_2_ZnSnSe_4_ phase and well matched to literature reports [4,8,35,45,52,53]. The most intense peak in the Raman spectrum of the tetragonal kesterite Cu_2_ZnSnSe_4_ phase is observed at 190 cm^−1^, which corresponds to the purely anionic vibrations (A1 mode) of selenium surrounded with motionless neighboring atoms [3,35,42]. Moreover, it can be appreciated that both Raman peaks are relatively broad in nature, which is attributed to the lattice defect-induced phonon confinement, low crystallinity, and the presence of strain within the nanostructure of CZTSe [35,42,51]. Moreover, the high purity, single phase, and quality of synthesized CZTSe is confirmed by the absence of impurity phases as no peaks occurred at 180 cm^−1^ (Cu_2_SnSe_3_) and 186 cm^−1^ (SnSe_x_) [4,35].

**Figure 2:**
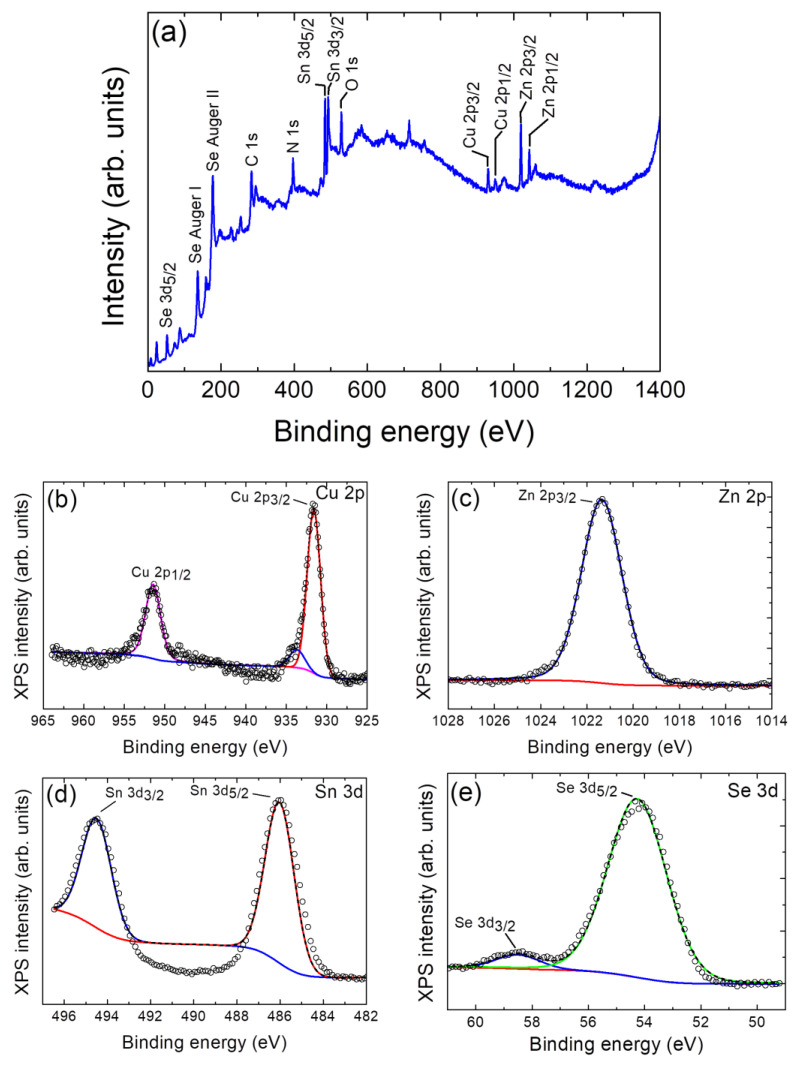
(**a**) X-ray photoelectron spectroscopy survey spectrum for the synthesized particulate Cu_2_ZnSnSe_4_ sample. XPS high-resolution spectra for (**b**) Cu 2p, (**c**) Zn 2p, (**d**) Sn 3d, and (**e**) Se 3d for the CZTSe-NPs. The short dashed black lines are the fit lines, while the colored lines represent each individual component. The original spectra are represented as small open circles.

The synthesized nanoparticulate Cu_2_ZnSnSe_4_ samples have been further analyzed by XPS to check the presence of Cu, Zn, Sn, and Se and to verify the valence states of these constituent elements. The full XPS spectrum of the sample in Figure 2a shows the peaks of Cu, Zn, Sn, and Se together with the C, N, and O peaks. It should be noted that the impurity peaks of C and O may be related to the reference or environmental contamination. The sharpened peaks shown in Figure 2a (actually at 136 eV and 177 eV) belong to Auger signals of Se, SeL2M45M45, and SeL3M45M45, respectively.

The valence state of elements in the CZTSe was evaluated by high-resolution XPS spectra, which are depicted in Figure 2b–e. High-resolution spectra for all the core levels of interest (Cu 2p, Zn 2p, Sn 3d, Se 3d) have been acquired. The spectra were calibrated at C 1s 284.8 eV. The surface signal, as it comes from the integral amplitudes in XPS, is 10.7% Cu, 28.7% Zn, 22.7% Sn, and 37.9% Se. According to the Cu 2p spectrum, a main peak is shown at 931.6 eV and a secondary one at 933.7 eV, which can be related to Cu^2+^ [54]. According to the Zn 2p spectrum, the main peak at 1021.4 eV corresponds to ZnO. According to the Sn 3d peaks, the mean peak appears at 486.0 eV, which is related to SnO_2_ [54] and possibly formed after thermal treatment. Finally, the Se 2p peaks show the main signal at 54.3 eV and correspond to selenides, while the minor peak at 58.5 eV corresponds to SeO_2_ [54].

**Figure 3:**
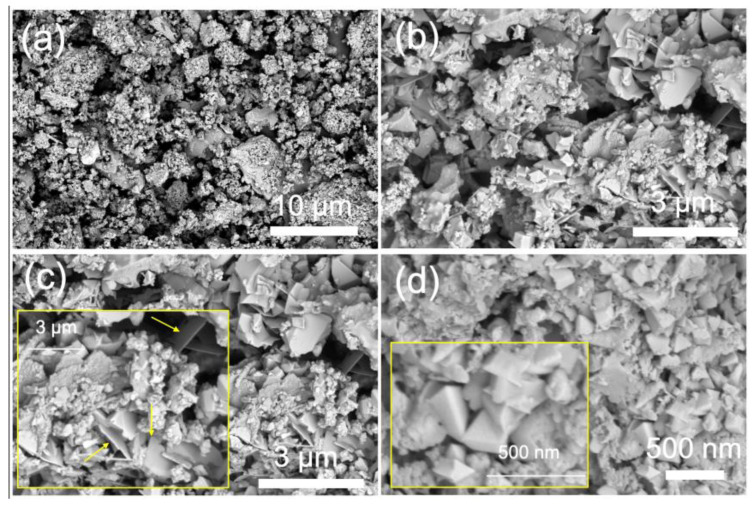
(**a**–**d**) FE-SEM plan-view micrograph images of a typical solvothermally synthesized nanoparticulate Cu_2_ZnSnSe_4_ sample at different magnifications. Higher magnification FE-SEM micrographs are depicted as insets of panels (**c**,**d**): the one in panel (**c**) shows the presence of sheet-like nanostructures (indicated by yellow arrows), and the other one in panel (**d**) highlights the presence of well-defined nanocrystals.

Field emission-scanning electron microscopy (FE-SEM) and transmission electron microscopy (TEM) were employed to precisely characterize the morphological and microstructural characteristics and chemical composition of CZTSe-NPs. Figure 3a,b depict low-magnification FE-SEM micrograph images of a typical solvothermally synthesized Cu_2_ZnSnSe_4_ sample. These figures show the grain growth structure and reveal two different morphologies. On the one hand, it can be appreciated (on the entire micrograph images) that the sample is made up of many mono-disperse nanocrystal agglomerates of irregular shape and with a uniform size distribution in the range of 200–400 nm. On the other hand, in certain regions, the presence of big CZTSe lumps of several microns can be observed (indicating a poor disaggregation process during the microscopy sample preparation); their growth is probably due to the aggregation of these very tiny above-mentioned agglomerates. Figure 3c,d depicts other FE-SEM plan-view micrograph images of this CZTSe sample. Figure 3c,d reveals the presence of nanocrystallites exhibiting two different hierarchical structures with sheet-like and pyramidal/tetragonal morphologies. The inset of Figure 3c displays some nearly vertical nanosheets, which disclose clearly that these sheets are very thin, and the average thickness is only about 40 nm. Similar nanoflake- and nanosheet-like structures in hydrothermal, solvothermal, and hot injection synthesized Cu_2_ZnSnSe_4_ nanocrystals have been previously observed and then reported in the literature [12,37,44]. Moreover, as has been said above, the presence of pyramidal/tetragonal nanocrystals of about 160 nm can be appreciated (see inset of Figure 3d). 

**Figure 4:**
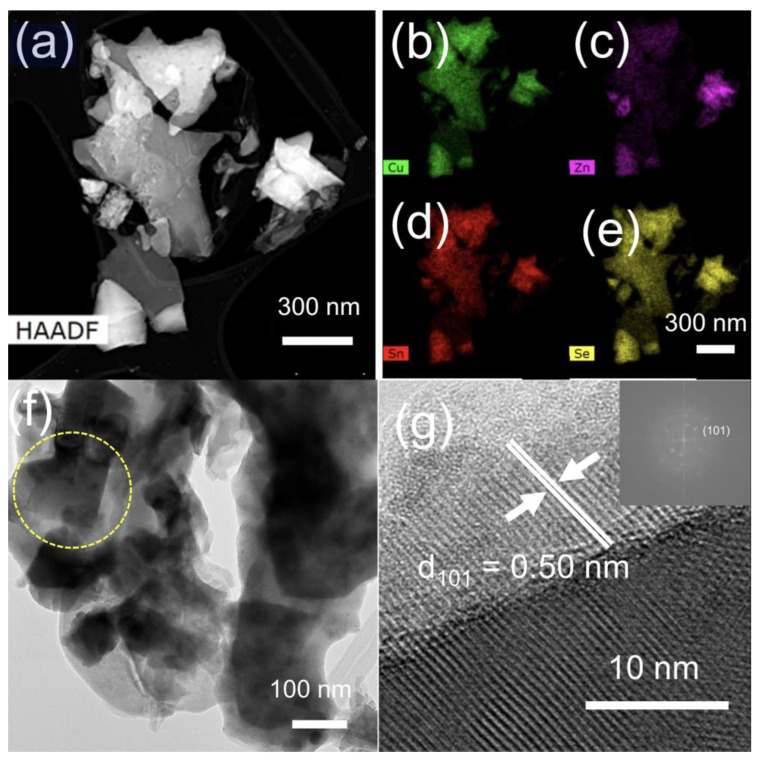
(**a**) TEM micrograph image of a typical nanoparticulate Cu_2_ZnSnSe_4_ cluster and (**b**–**e**) its corresponding EDX elemental mapping for Cu (green), Zn (magenta), Sn (red), and Se (yellow), as indicated. (**f**) High-magnification TEM micrograph of a typical nanoparticulate Cu_2_ZnSnSe_4_ cluster. The presence of square-shaped nanocrystals with defined and sharp edges can be appreciated, and one of them is highlighted by a yellow dotted circle. (**g**) HRTEM micrograph of a single CZTSe nanocrystal. Lattice fringes corresponding to the (101) crystallographic planes of the tetragonal crystalline structure of Cu_2_ZnSnSe_4_ have been highlighted. Inset: The Fast Fourier Transform pattern was obtained from the HRTEM image.

Figure 4a shows a TEM micrograph image of a typical nanoparticulate Cu_2_ZnSnSe_4_ sample, where the presence of an agglomeration of CZTSe nanocrystals with irregular shapes can be appreciated. Moreover, to confirm the presence of all four elements in the nanopowder sample, EDS elemental mapping was conducted, demonstrating the presence of Cu, Zn, Sn, and Se elements homogeneously distributed in the whole CZTSe nanopowder sample, as shown in Figure 4b–e. Figure 4f shows a high-magnification TEM micrograph image of a typical Cu_2_ZnSnSe_4_ sample. Fine nanoparticles of ca. 90 nm in size can be observed along with their agglomerates of ca. 600 nm. This agglomerate is constituted by both round and square-shaped nanocrystals (this last type is indicated by a yellow dotted circle). The finer details of the microstructure of the CZTSe nanoparticles were further investigated by high-resolution transmission electron microscopy (HRTEM), which is shown in Figure 4g. The HRTEM micrograph image of a single CZTSe nanocrystal, shown in Figure 4g, indicates that the nanoparticles are crystalline in nature and exhibit lattice fringes with an interplanar distance of 0.50 nm, which matches well with the interplanar distance of the (101) plane of the tetragonal crystalline structure of Cu_2_ZnSnSe_4_ [48]. The Fast Fourier Transform pattern of the HRTEM image (inset of Figure 4g) is also consistent with the tetragonal CZTSe phase. 

### 3.2. Surface Area of the Nanoparticulate Cu_2_ZnSnSe_4_ Samples

**Figure 5:**
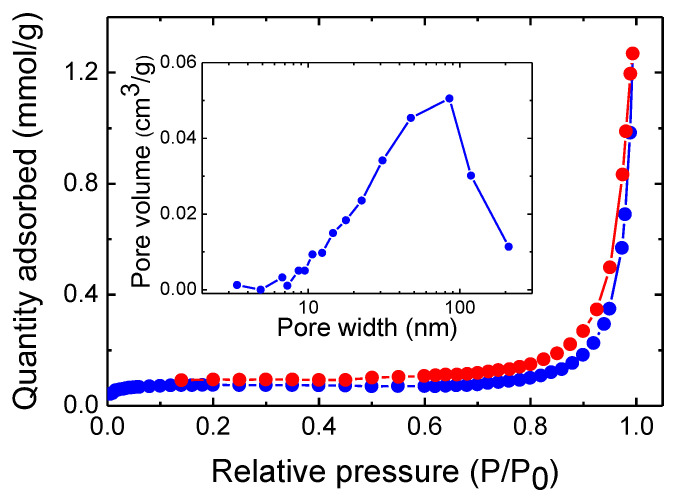
Nitrogen adsorption and desorption isotherms (blue and red symbols, respectively) at −196 °C for a typical nanocrystalline Cu_2_ZnSnSe_4_ sample. Inset: Pore size distribution of this Cu_2_ZnSnSe_4_ sample.

To study the porous nature of the nanoparticulate Cu_2_ZnSnSe_4_ samples, nitrogen adsorption–desorption isotherms have been performed at −196 °C after outgassing the sample at 180 °C for 6 h; the results are shown in Figure 5. From the graph, it can be seen that the adsorption–desorption isotherm of the CZTSe sample exhibits a hysteresis loop-like nature, which is the main feature of the porous materials [32]. According to the Brunauer–Deming–Deming–Teller (BDDT) classification, the majority of physisorption isotherms can be grouped into six types [55,56]. Moreover, the shapes of the hysteresis loops are often used to identify the specific pore structure [55,56]. It can be appreciated that according to this classification, the CZTSe sample displays a Type IV isotherm and a Type H1 hysteresis loop [55,56]. The characteristic ¨hysteresis loop¨ of the Type IV systems is indicative of the presence of narrow pores that facilitate capillary condensation [55,56]. It must be pointed out that Type IV isotherms are given by many mesoporous industrial adsorbents and catalysts [55,56]. Moreover, Type H1 is often associated with porous materials consisting of agglomerates or compacts of approximately uniform nanoparticles in a fairly regular array, hence exhibiting a narrow distribution of pore size [56]. The specific surface area of these samples was obtained using the Brunauer–Emmet–Teller (BET) gas adsorption method [55,56]. The quaternary material exhibits a BET surface area of ca. 7 m^2^/g, lower than the previously reported surface area of hydrothermally synthesized CZTSe nanocrystals of 15 m^2^/g [57]. The pore size distribution for this CZTSe sample is depicted as an inset in Figure 5, showing a broad maximum at 60–90 nm, indicating the presence of macropores [56].

### 3.3. Optoelectronic Characterization

The optoelectronic properties of the nanoparticulate Cu_2_ZnSnSe_4_ samples, i.e., optical and semiconducting properties, have been verified through optical UV–visible absorption spectrometry measurements and by Mott–Schottky analysis, respectively.

**Figure 6:**
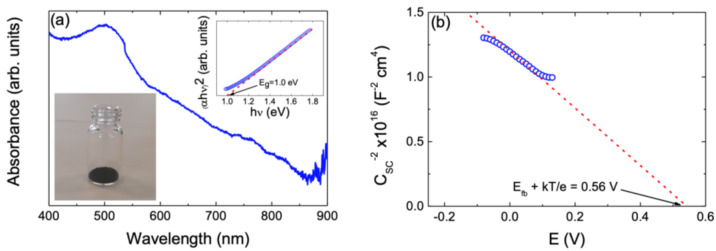
(**a**) UV–visible optical absorbance spectrum of a typical nanocrystalline Cu_2_ZnSnSe_4_ sample. The lower inset of (**a**) depicts a digital photograph of CZTSe nanocrystal powder. The upper inset shows the Tauc plot for *E_g_* determination and corresponding linear fitting (red dashed line). (**b**) Mott–Schottky plot for a synthesized particulate Cu_2_ZnSnSe_4_ sample. The red dashed line represents the fitted data in the linear range of the curve. The intercept of this line with the x-axis determines the flat band potential (*E_FB_*) as indicated. Measurements were carried out in 0.1 M Na_2_SO_4_ (pH = 6.5) with an AC frequency of 10 kHz.

One of the most critical parameters in predicting the photocatalytic performance of a semiconductor is the determination of the optical bandgap energy (E_g_). For this purpose, UV–visible spectrophotometry was employed to analyze the CZTSe absorption spectrum. Figure 6a depicts the optical absorption spectrum of a typical nanoparticulate Cu_2_ZnSnSe_4_ sample. As shown in Figure 6a, CZTSe nanoparticles absorb light over the entire range of the visible light wavelength, and the absorbance even extends to larger wavelengths. So, this broad and strong optical absorbance makes this material an advantageous and suitable candidate for photocatalytic applications. The E_g_ and optical transition type of nanoparticulate Cu_2_ZnSnSe_4_ samples have been determined from the Stern relation of near-edge absorption [20,50,58,59] (please see SI).

The inset in Figure 6a shows the Tauc plot graph of (αhν)^2^ against *hν* so the optical bandgap can be determined. Equation (S3) may be used to fit the optical absorption in the edge region of all nanoparticulate Cu_2_ZnSnSe_4_ samples. This indicates that a direct allowed transition is responsible for the absorption edge, which is consistent with other findings that have been documented in the literature [4,35,42,45,53,60,61]. A direct energy optical E_g_ of ca. 1.0 eV has been extracted by extrapolating the dashed line in the inset of Figure 6a to the energy axis. This value agrees very well with previously literature-reported bandgap values of 0.8–1.0 eV [4,35,42,45,53,60,61].

Moreover, a Mott–Schottky analysis was performed to establish the characteristics of the quaternary phase of CZTSe as a semiconductor material (see Figure 6b). This plot representation allows the flat band potential to be determined while the carrier concentration and the type of conductivity can be obtained from the plot slope value. The Mott–Schottky analysis was carried out in a potential region around 0 V, where faradaic processes are not observed. Under these conditions, the total capacitance, *C*, can be approximated by the semiconductor capacitance (*C_sc_*) in the depletion region. This assumption is valid at a low carrier concentration. The *C_sc_* of the semiconductor with the potential depends on it according to the following equation [62,63,64,65,66]:

(1) $$\frac{1}{C_{sc}^{2}}=\frac{2}{e\varepsilon \varepsilon_{0}N_{A}A^{2}}[\left(-E+E_{FB}\right)-\frac{kT}{e}]$$

where *e* is the charge of the electron (1.6 × 10^−9^ C), *N_A_* is the acceptor concentration for a p-type semiconductor, *ε* is the dielectric constant of CZTSe, *ε*_0_ is the permittivity of vacuum (8.85 × 10^12^ F/m), *E_FB_* is the flat band potential, *T* is the absolute temperature (300 K), *k* is the Boltzmann constant (1.38 × 10^−19^ J/K), and *A* is the surface area of the electrode (1.5 cm^2^). The clear negative slope observed in Figure 6b demonstrates that the CZTSe phase presents a p-type behavior characterized by a low concentration of charge acceptors (*N_A_*). The concentration of charge acceptors has been calculated (considering a value of *ε* = 8.6) to be 1.38 × 10^−16^ cm^−3^ [67]. On the other hand, a flat band potential value (*E_FB_*) of 0.56 V (*E_FB_ = E + kT/e*) can be obtained through the extrapolation shown in Figure 6b. These results are consistent with those reported in the literature [68,69].

### 3.4. Electrochemical Impedance Spectroscopy (EIS) Analysis

**Figure 7:**
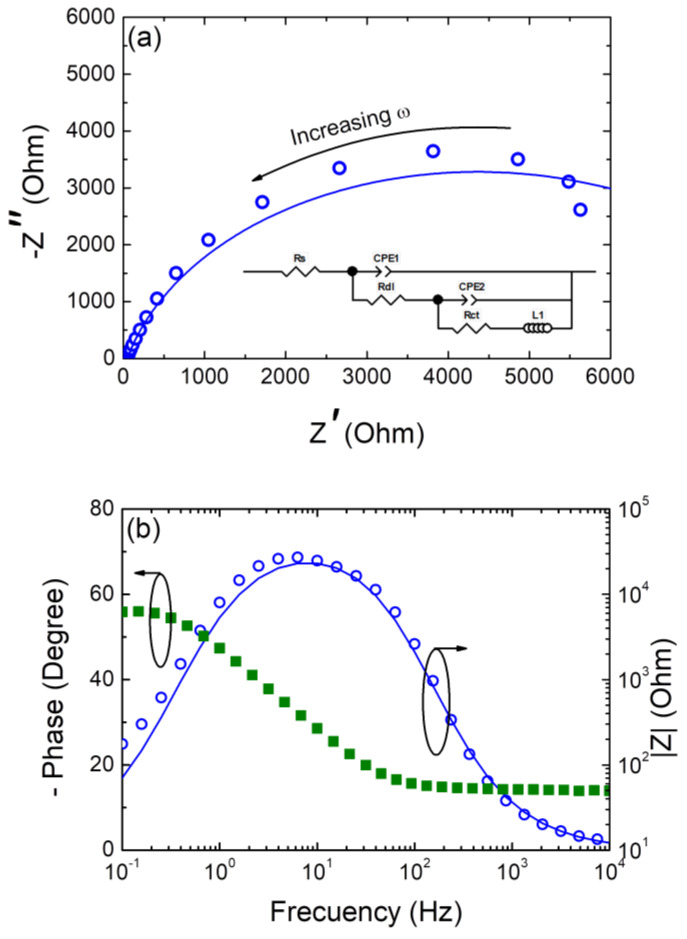
(**a**) Nyquist and (**b**) Bode plots from the electrochemical impedance spectroscopy (EIS) study of the nanoparticulate CZTSe sample. Inset of panel (**a**) shows the equivalent electronic circuit model used to fit the experimental EIS data. The blue line and the green block are the fitting result with the electrical circuit model. Measurements were carried out in a 0.1 M Na_2_SO_4_ electrolytic solution under open circuit potential conditions.

To further probe the electrocatalytic performance of the nanoparticulate CZTSe samples, electrochemical impedance spectroscopy (EIS) measurements were carried out, and the corresponding Nyquist and Bode diagrams are depicted in Figure 7. The Nyquist plot was then fitted with an equivalent electrical circuit (see inset of Figure 7a), and the interpreted data are shown in Table 1. The proposed equivalent circuit considers the solution resistance (R_s_) and two constant phase shift elements (CPE)/R couples. The last is due to the surface irregularity due to the drop-casting electrode assembly process. The CPE_1_/R_dl_ couple represents the capacitance of the electrical double layer, while the CPE_2_/R_ct_ couple corresponds to the transfer of electrons at the semiconductor/electrolyte Na_2_SO_4_ interface. In addition, an inductor element was incorporated to model the response obtained in the low-frequency region of the spectra. The fit indicated by a solid line in the plot showed good agreement with the data; this confirmed the suitability of the equivalent circuit model employed. Capacitive behavior can be observed without the presence of a diffusional process, unlike what was observed by other authors [70,71,72]. In fact, the alpha values close to 1, as shown in Table 1, confirm this behavior. Moreover, the low R_ct_ obtained to the fit indicates a high oxidizing power of the hole in the valence band of the semiconductor material, being able to capture an electron from the aqueous solvent with the subsequent formation of the hydroxyl radical. Finally, the deviation at the low-frequency region may be related to a surface change resulting from a minimum detachment of the CZTSe phase from the electrode surface with immersion time in the electrolyte.

### 3.5. Photocatalytic Activity Evaluation

**Figure 8:**
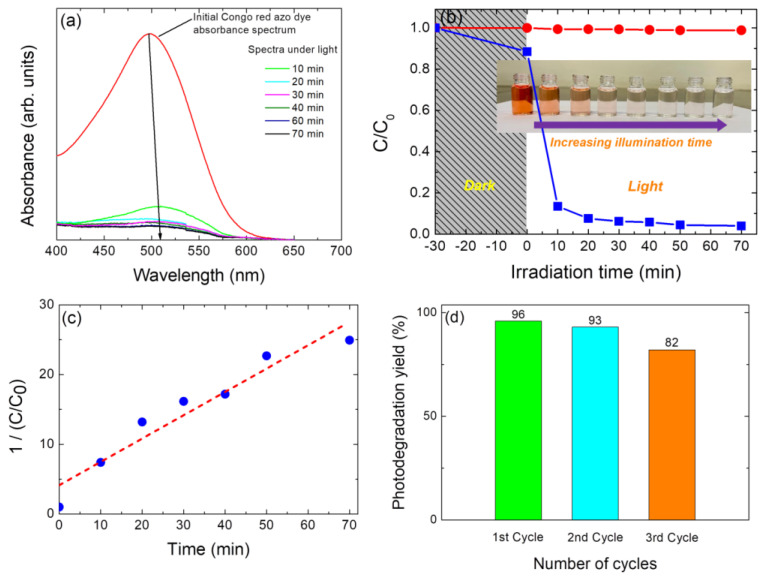
Photocatalytic degradation of Congo red azo dye in aqueous solution using CZTSe-NP photocatalyst under AM1.5G simulated sunlight (100 mW cm^−2^) illumination. (**a**) Absorbance spectra as a function of illumination time, demonstrating the photodegradation of CR dye against CZTSe. (**b**) Relative concentration (*C*/*C*_0_) versus time for photodegradation of CR azo dye in the presence (square blue symbols) and in the absence (red round symbols) of CZTSe-NP photocatalyst under the same illumination conditions used in (**a**). The inset shows the photographs of photocatalytic degradation of CR azo dye, from left to right: from 0 min to 70 min of illumination time, at 10 min intervals between samples. (**c**) Pseudo-second-order kinetic plot for the photocatalytic degradation of Congo red azo dye. (**d**) Recyclability of the nanoparticulate CZTSe sample as a photocatalyst for the photodegradation of CR azo dye.

The photocatalytic performance of the nanoparticulate Cu_2_ZnSnSe_4_ samples was tested as a proof of concept by measuring the amount of Congo red azo dye that degraded in an aqueous solution under 100 mW cm^−2^ of AM1.5G simulated sunlight. The dye aqueous solution’s optical absorption spectrum (before light on) and the time-dependent absorbance spectra of the dye aqueous solution’s photodegradation—which were recorded in the 400–700 nm wavelength range—are both displayed in Figure 8a. Owing to distinctive optical absorption associated with the n-π* transition of the lone pair on the N atom of the azo chromophore (-N=N-), a distinctive peak at 497 nm is seen [73]. It can be appreciated that this characteristic optical absorption peak diminished with illumination time (indicating that the concentration of Congo red dye decreases). Figure 8b shows the relative concentration (C/C_0_) changes of CR dye versus the solar simulator irradiation time. To validate the role of synthesized nanoparticulate Cu_2_ZnSnSe_4_ photocatalyst, controlled experiments have also been carried out in the absence of photocatalyst, though no significant degradation of CR has been observed (see Figure 8b, red line symbols). Figure 8b shows that the photocatalytic activity of nanoparticulate Cu_2_ZnSnSe_4_, after 70 min illumination, reaches a 96% degradation efficiency value. In previously published studies and employing different semiconductor photocatalysts such as TiO_2_, CdS, or Fe_3_O_4_, Congo red dye removal yields greater than 90% are achieved after 200 min under UV light illumination. On the other hand, in the present case, a superior removal performance of greater than 90% is achieved only in 70 min and under a solar simulator light illumination [74,75,76,77]. This suggests a structural modification of the CR dye due to a photo-oxidative process either by the action of holes in the valence band of CZTSe or by its reaction with the formed hydroxyl radicals [33]. 

The kinetics of the photodegradation process have been further studied by using the pseudo-second-order kinetic. The pseudo-second-order equation is expressed in its integrated linear form as follows [78,79]:

(2) $$\frac{1}{\left[C_{t}\right]}=\frac{1}{\left[C_{0}\right]}kt$$

where *C*_0_ is the initial concentration of Congo red azo dye, *C_t_* is the concentration of this dye at time *t*, *t* is the contact time in minutes, and *k* is the second-order rate constant. As shown in Figure 8c, the variation of CR concentration with time could be adjusted considering a pseudo-second-order model through Equation (2), obtaining a rate constant *k* = 0.334 min^−1^ (with a regression of 0.963). It should be noted that this is the first value reported employing a CZTSe and that it establishes an initial standard for its evaluation as a heterogeneous photocatalyst. On the other hand, when comparing the CZTS and CZTSe phases under the same experimental conditions (mass of photocatalyst and illumination power), a change in the kinetic model can be observed from pseudo-first-order CZTS to pseudo-second-order for CZTSe characterized by a rate constant of 0.01 min^−1^ and 0.334 min^−1^, respectively [33]. The change in the kinetic model would imply a greater generation of hydroxyl radicals that attack CR, with the CZTSe phase being more efficient than in the case of CZTS one.

Furthermore, a reusability study of the quaternary CZTSe photocatalyst in the degradation of Congo red azo dye has been performed. To this end, the photocatalyst has been reused by washing it several times with distilled water to ensure the absence of adsorbed pollutant molecules on the kesterite surface. No significant reduction in the photocatalytic activity of CZTSe photocatalyst has been observed for two consecutive trials (96% to 93%); however, in the third one, a greater decrease can be observed (96% to 82%), which can be attributed to a photocatalyst mass loss during the successive washing steps for reuse. Then, the merits of the CZTSe lie in the reusability property of the photocatalyst for the redemption and degradation of Congo azo dye. Similar results have been previously reported for Rhodamine B (RhB) with the CZTSe phase, even at a higher number of cycles [16,35]. Furthermore, it is important to highlight that the present study represents, to our knowledge, the first time that this photocatalyst has been used to degrade Congo red azo dye, as no previous studies have been conducted on the photocatalytic degradation of this dye employing Cu_2_ZnSnSe_4_ quaternary compound.

## 4. Conclusions

In the present study, Cu_2_ZnSnSe_4_ nanoparticles have been successfully prepared via a simple, cost-effective, and green solvothermal method without surfactants or templates. The XRD results indicate that the formed kesterite CZTSe phase is well-defined, and the samples are polycrystalline after 72 h of solvothermal reaction, followed by an annealing process. Raman peaks at 173 and 190 cm^−1^ confirm the formation of a pure CZTSe phase. Moreover, the high purity, single phase, and quality of synthesized CZTSe are confirmed by the absence of impurity phases, as no peaks occurred at 180 cm^−1^ (Cu_2_SnSe_3_) and 186 cm^−1^ (SnSe_x_). XPS results revealed the oxidation states as Cu^2+^, Zn^2+^, Sn^4+^, and Se^2−^. TEM micrograph images revealed the presence of nanoparticles with average sizes of ~90 nm. A BET surface area of 7 m^2^/g has been determined. The CZTSe NPs showed a bandgap of 1.0 eV and a p-type semiconducting behavior. Furthermore, the EIS responses show a low resistance to charge transfer, confirming the oxidizing power of the hole in the valence band of CZTSe. The Cu_2_ZnSnSe_4_ nanoparticle photocatalyst under simulated sunlight results in almost complete degradation (96%) of CR dye after 70 min, following a pseudo-second-order kinetic model (rate constant of 0.334 min^−1^). Outcomes are superior to those of other photocatalysts, like TiO_2_, which is well known for its high efficiency. After three runs of the photodegradation process, the reusability experiment verifies the stability of the photocatalytic activity of the as-prepared CZTSe nanopowder.

## Figures and Tables

**Table 1:** Equivalent circuit element values obtained through a complex non-linear least-squares fitting of the EIS spectra ^a^.

R_s_ (Ω)	CPE_1_		R_dl_ (Ω)	CPE_2_		R_ct_ (Ω)	L_1_ (H)
	Q_1_ ^b^ (s^α1^Ω^−1^)	α_1_		Q_2_ ^b^ (s^α2^Ω^−1^)	α_2_		
50.08	5.94 × 10**^−^**^5^	0.9	3 × 10^3^	9.654 × 10**^−^**^5^	1.0	5.634 × 10^3^	38.07

^a^ See inset of Figure 7a for the involved electrical equivalent circuit. ^b^ The impedance of a CPE is defined as $Z_{CPE}=\frac{1}{{Q(jw)}^{\alpha }}$, where α (0 < α < 1) is an empirical constant with no real physical meaning.

## Data Availability

Data are contained within the article.

## Supplementary Materials

The following supporting information can be downloaded at: https://www.mdpi.com/article/10.3390/nano14131079/s1; we present the details of the experimental conditions that were used for the characterization and evaluation of CZTSe-NPs as a photocatalyst. It also contains some conceptual aspects for the determination of the crystallite size and the optical E_g_ of the semiconductor.
