# Supplementary material for: Solvothermal Synthesis of Cu2ZnSnSe4 Nanoparticles and Their Visible-Light-Driven Photocatalytic Activity
Source: Nanomaterials (Basel). 2024 Jun 24;14(13):1079. doi: 10.3390/nano14131079 (PMC11243532; doi:10.3390/nano14131079)
Supplement: Supplementary file 1 [file nanomaterials-14-01079-s001.zip › nanomaterials-3045551-supplementary.pdf]

SUPPLEMENTARY MATERIAL

Solvothermal synthesis of $Cu_2ZnSnSe_4$ nanoparticles and their visible-light-driven photocatalytic activity

Rodrigo Henríquez [1,*], Paula Salazar Nogales [1], Paula Grez Moreno [1], Eduardo Muñoz Cartagena [1], Patricio Leyton Bongiorno [1], Pablo Zerega Garate [1], Elena Navarrete-Astorga [2], Enrique A. Dalchiele [3]

[1] Instituto de Química, Facultad de Ciencias, Pontificia Universidad Católica de Valparaíso, Casilla 4059, Valparaíso 2340000, Chile; paula.salazar@pucv.cl (P.S.N.); paula.grez@pucv.cl (P.G.M.); eduardo.munoz.c@pucv.cl (E.M.C.); patricio.leyton@pucv.cl (P.L.B.); pablo.zerega@pucv.cl (P.Z.G.)
[2] Laboratorio de Materiales y Superficie, Departamento de Física Aplicada I, Universidad de Málaga, 29071 Málaga, Spain; enavarrete@uma.es
[3] Instituto de Física, Facultad de Ingeniería, Herrera y Reissig 565, C.C. 30, Montevideo 11000, Uruguay; dalchiel@fing.edu.uy
*Correspondence: rodrigo.henriquez@pucv.cl; Tel.: +56-32-2274921

- *Characterization of the CZTSe-NPs*

The structural characterization of the CZTSe phase was performed by X-ray diffraction (XRD) using a Bruker D8 ADVANCE diffractometer. The operating conditions were: CuKα radiation (30 mA, 40 kV, λ= 0.15418 nm), in Bragg-Brentano 45° mode, with a step of 0.01° and a step time of 34 s at room temperature.

Raman spectra were obtained using a Witec Alpha 300 confocal Raman microscope system equipped using an excitation laser wavelength of 785 nm and an electrically cooled CCD camera. The signal was calibrated using the 520 cm^{-1} line of a Si wafer and a 20x objective. The laser power on the samples was 2 mW. The resolution was set to 4 cm^{-1} and 10 scans with an integration time of 1 second were performed. Peak parameters have been obtained by least square Lorentz fitting using Origin 8.0 software.

The chemical composition of the $Cu_2ZnSnSe_4$ samples was studied via X-ray photoelectron spectroscopy (XPS) using a Physical Electronics (PHI) VersaProbe II spectrometer equipped with an Al Kα radiation source (1486.6 eV and 47.3 W).

Field emission scanning electron microscopy (FE-SEM) images of the CZTSe samples were obtained on a Helios Nanolab 650 Dual Beam equipment from FEI Company. For this, the powders were supported on a conductive carbon tape inside the vacuum chamber. The analysis of the chemical composition of the formed structures was carried out using X-ray energy dispersion spectrometry (EDS). The equipment used was a QUANTAX 200 model from Bruker with XFLASH (EDS coupled to a SEM equipment: Hitachi SEM SU-3500 of variable pressure with a detector 410-M). Samples for TEM were ultrasonically dispersed in 1 ml of ethanol. A small drop of the suspended solution was placed on a porous carbon film on a nickel screen and allowed to air dry. Transmission electron

microscopy (TEM) and High-resolution transmission electron microscopy (HRTEM) images were obtained on a Talos F200X instrument.

Optoelectronic properties of the nanoparticulate $Cu_2ZnSnSe_4$ samples, i.e.: optical and semiconducting properties have been verified through optical UV-visible absorption spectrometry measurements and by Mott-Schottky analysis, respectively, as it will be detailed below.

The optical properties were studied by UV-VIS molecular absorption spectra through the transmittance spectrum, using a SHIMADZU UV-2600 Spectrophotometer with a PC connection. The measurement range was from 400 nm to 900 nm at room temperature, with a scanning speed of 0.2 nm s^{-1}, 10 mg of CZTSe nanoparticles suspended in ethanol were prepared.

- *Photodegradation of CR azo dye and photocatalyst regeneration studies*

The solution of CR azo dye with the CZTSe-NP was shaken in the dark for 30 min to generate adsorption-desorption equilibrium. Then, the solution was exposed to an ABET Technologies Model 11002 SunLite solar simulator (1000 W), and photodegradation was examined by monitoring the absorption spectra of CR azo dye solutions after various irradiation times using a Shimadzu UV-2600 UV-Vis spectrophotometer. The measurement wavelength range was 400-700 nm at room temperature, with a scanning speed of 0.2 nm s^{-1}. Prior to photodegradation, a calibration curve of the CR dye was made, and the following equation was used to determine the degradation efficiency:

$$Degradation\ efficiency\ (\%) = \frac{C_0 - C_t}{C_0} \times 100 \quad \text{(S1)}$$

where C_0 corresponds to the initial concentration of Congo red azo dye prior to exposure to illumination and C_t corresponds to the concentration of CR dye after being exposed to solar illumination for a certain time t.

The cyclability of the CZTSe photocatalyst has been evaluated maintaining the experimental parameters described above. After each cycle, the nanocomposite was collected and washed with ethanol multiple times, dried for 30 min at 60°C, and then reused for subsequent runs. Naturally the loss of a portion of the photocatalyst has been observed, and then the amount of CZTSe photocatalyst used in each cycle was not maintained.

- *Crystallite size determination through the Scherrer equation*

The average crystallite size was calculated from the full width at half maximum (FWHM) of XRD peaks by using the well-known Scherrer formula [1,2]:

$$D = \frac{k\lambda}{\beta cos\theta} \quad \text{(S2)}$$

where D is the crystallite diameter, λ is the wavelength of the incident radiation, k = 0.89 is the shape factor, θ is the Bragg angle, and β is the full width at half maximum (FHWM) in radians.

- *Bandgap energy determination*

The bandgap (E_g) and optical transition type of nanoparticulate $Cu_2ZnSnSe_4$ samples have been determined from the Stern relation of near-edge absorption which is given as [2-5]:

$$\alpha = \frac{A_0\left(h\nu - E_g\right)^n}{h\nu} \qquad \text{(S3)}$$

where A_0 is a parameter related to the effective masses associated with the valence and conduction bands, and $h\nu$ is the photon energy (where ν is the frequency, h is the Planck's constant). m depends on the nature of band transitions, i.e.: n = 1/2 or 2 for direct or indirect allowed transitions, respectively.

REFERENCES

1. Cullity, B.D. Elements of X-Ray Diffraction, 2nd Edition. *Addison-Wesley Publishing Co. Reading MA* **1978**, 100-105,277-279.
2. Henríquez, R.; Vásquez, C.; Muñoz, E.; Grez, P.; Martín, F.; Ramos-Barrado, J.R.; Dalchiele, E.A. Phase-Pure Iron Pyrite (FeS_2) Micro- and Nano-Sized Crystals Synthesized by Simple One-Step Microwave-Assisted Hydrothermal Method. *Physica E Low Dimens Syst Nanostruct* **2020**, *118*, 113881, doi:https://doi.org/10.1016/j.physe.2019.113881.
3. Kisch, H. *Semiconductor Photocatalysis: Principles and Applications*; Wiley-VCH, 2015; ISBN 978-3-527-33553-4.
4. Pankove, J.I.; Kiewit, D.A. Optical Processes in Semiconductors. *J Electrochem Soc* **1972**, *119*, 156Ca, doi:10.1149/1.2404256.
5. Kumarage, W.G.C.; Wijesundera, R.P.; Seneviratne, V.A.; Jayalath, C.P.; Dassanayake, B.S. Tunable Optoelectronic Properties of CBD-CdS Thin Films via Bath Temperature Alterations. *J Phys D Appl Phys* **2016**, *49*, 95109, doi:10.1088/0022-3727/49/9/095109.
